# Platelet count and sleep quality in immune thrombocytopenia: correlation with 5-hydroxytryptamine and therapeutic implications of platelet-5-HT-melatonin axis dysregulation

**DOI:** 10.3389/fneur.2025.1645796

**Published:** 2025-10-20

**Authors:** Yingying Jiao, Wenxuan Fan, Yu Wang, Yuehong Qin, Yujiao Zhang, Jieyu Ye

**Affiliations:** Department of Hematology, Nanfang Hospital, Southern Medical University, Guangzhou, China

**Keywords:** immune thrombocytopenia, sleep quality, platelet count, 5-hydroxytryptamine, quality of life, Pittsburgh Sleep Quality Index

## Abstract

**Background:**

Immune thrombocytopenia (ITP) disrupts platelet homeostasis via autoimmune mechanisms, yet its systemic effects beyond bleeding risk remain poorly characterized. Sleep disturbances are frequently reported in ITP patients, but the relationship between thrombocytopenia, serotonergic signaling, and sleep architecture remains undefined. This study investigates whether platelet dynamics modulate sleep quality through the “platelet-5-HT-melatonin axis” and explores therapeutic implications of this axis in ITP.

**Methods:**

In a prospective longitudinal cohort of 87 ITP patients (baseline platelet count: 28.7 ± 15.2 × 10^9^/L) and 71 healthy controls, sleep quality was evaluated using the Pittsburgh Sleep Quality Index (PSQI) at baseline and after 12 weeks of standard glucocorticoid therapy. Subgroup analysis stratified patients by platelet count (<50 × 10^9^/L vs. ≥50 × 10^9^/L). Circulating 5-hydroxytryptamine (5-HT) levels were quantified in 42 patients via ELISA. Longitudinal changes in sleep metrics and 5-HT were analyzed using linear mixed-effects models (LMMs), with Cohen’s d effect size to assess clinical relevance. Correlations between platelet count, PSQI scores, and inflammatory markers (hs-CRP, IL-6) were explored.

**Results:**

At baseline, ITP patients had significantly higher PSQI scores than controls (12.4 ± 3.8 vs. 6.2 ± 2.1, *p* < 0.001), mainly reflecting impaired efficiency (*d* = 0.89), prolonged latency (*d* = 0.75), and daytime dysfunction (*d* = 0.92). Platelet count inversely correlated with PSQI (*r* = −0.223, *p* = 0.04), and 68% of patients with <50 × 10^9^/L platelets had severe sleep disturbance (PSQI >10). After treatment, patients achieving platelet normalization (>100 × 10^9^/L) showed significant improvements in sleep latency (−23.5%, *p* = 0.008), duration (+18.2%, *p* = 0.02), and 5-HT levels (+42.7%, *p* < 0.001), alongside reduced fatigue scores. Mechanistically, thrombocytopenia impaired 5-HT uptake and reduced nocturnal melatonin secretion, while elevated hs-CRP and IL-6 indicated inflammation-driven hypothalamic-pituitary-adrenal axis dysregulation.

**Conclusion:**

ITP-associated thrombocytopenia disrupts sleep architecture through serotonergic dysregulation and chronic inflammation. Standard glucocorticoid therapy not only restores platelet counts but also ameliorates sleep quality and fatigue, highlighting the therapeutic potential of platelet-targeted interventions for comorbid sleep disorders. These findings advocate for routine sleep assessment in ITP management and integration of 5-HT-modulating strategies into comprehensive care frameworks to address the hematologic-immunologic-sleep axis.

## Graphical abstract

Improvement of sleep quality in ITP patients and its association with treatment efficacy.
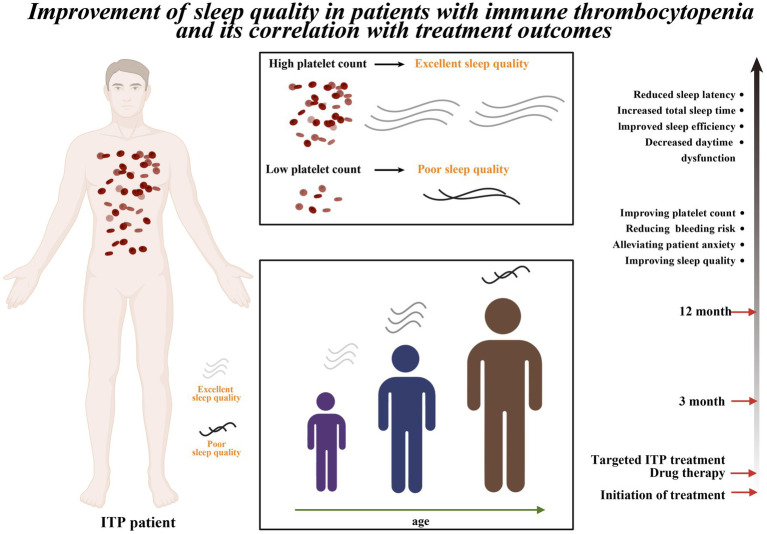

## Introduction

Immune thrombocytopenia (ITP) is an autoimmune disorder characterized by increased platelet destruction and impaired platelet production, leading to reduced peripheral blood platelet counts (1). The pathophysiology, clinical manifestations, and treatment responses of ITP exhibit significant heterogeneity (2–7). The immune dysregulation in ITP not only causes thrombocytopenia but also triggers a persistent low-grade systemic inflammatory response through the activation of pro-inflammatory cytokine networks, such as interleukin-6 (IL-6) and tumor necrosis factor-α (TNF-α) (1, 6).

Chronic inflammation may disrupt sleep–wake rhythms via mechanisms including hypothalamic-pituitary-adrenal (HPA) axis dysfunction and disturbances in 5-hydroxytryptamine (5-HT) and melatonin metabolism (8). Similar to other autoimmune diseases such as rheumatoid arthritis and systemic lupus erythematosus, multiple studies have demonstrated impaired sleep quality in ITP patients (9–12). As understanding of ITP deepens, growing research has focused on its impact on patients’ quality of life (1). The ITP World Impact Survey (I-WISH) demonstrated that ITP patients exhibit significantly impaired health-related quality of life (HRQOL) compared to healthy populations (13, 14). This negative impact extends beyond physical health to include psychological and emotional well-being (15–17). Studies indicate that approximately 25% of ITP patients require pharmacological assistance to improve sleep quality (18, 19), and their sleep disorders frequently correlate with the severity of thrombocytopenia (20). However, current international consensus reports and ASH guidelines only sporadically mention ‘sleep/mood’ issues within the context of adverse effect monitoring, lacking systematic intervention recommendations (21, 22). This finding underscores the importance of considering sleep quality in ITP treatment and management.

Adequate sleep is crucial for maintaining both physical and mental health, serving as a fundamental basis for improving quality of life (23–25). The 2019 American Society of Hematology guidelines for ITP treatment emphasize that therapeutic goals should not only focus on preventing bleeding, maintaining appropriate platelet counts, and minimizing treatment-related toxicity but, more importantly, on enhancing patients’ quality of life (22). While this objective has gained broad recognition, current assessments of sleep health’s impact on ITP patients’ quality of life remain relatively insufficient (26–28). Existing studies predominantly consist of cross-sectional surveys with limited longitudinal data to verify the causal implication of whether “changes in platelet counts can reverse sleep disorders.” Furthermore, there is a lack of integrated exploration into potential inflammatory-neuroendocrine pathways. To address these research gaps, this study systematically investigated sleep quality in ITP patients using the Pittsburgh Sleep Quality Index (PSQI) (29, 30), aiming to contribute to this underexplored field.

In this study, the PSQI was employed as the primary assessment tool in a 12-week follow-up of ITP patients to delineate the dynamic relationship between platelet counts and sleep quality, and to explore the potential mediating role of 5-HT. Although the COVID-19 pandemic may have influenced sleep patterns in the general population, rigorous multi-stage screening, including mental health evaluation, was applied to minimize non-disease confounding. We hypothesized that (i) sleep disturbances in ITP exhibit a threshold effect of platelet counts, and (ii) such disturbances are predominantly driven by chronic inflammation (IL-6) and dysregulation of the 5-HT/melatonin axis, with platelet counts serving only as a surrogate marker of inflammatory burden. These findings are expected to inform the integration of sleep health interventions into comprehensive ITP management.

## Materials and methods

### Study subjects and sample selection

This study was designed as a prospective, single-center longitudinal cohort. Recruitment started in June 2020, and baseline assessments were completed by December 2021. All participants were followed until March 2022. The study population included patients with ITP treated at the Department of Hematology, Nanfang Hospital, Southern Medical University. The exposure of interest was a confirmed diagnosis of ITP (based on the 2019 IWG consensus criteria), and the outcome measure was sleep quality assessed by the PSQI.

Inclusion criteria for patients were: (1) age 18–68 years; (2) diagnosis of primary ITP according to IWG standards. Exclusion criteria were: (1) secondary thrombocytopenia; (2) cognitive impairment, psychiatric disorders, or other malignant diseases; (3) diagnosis of anxiety or depressive disorders according to DSM-5; (4) HAMA score >14 or PHQ-9 score >10; (5) use of antidepressants or benzodiazepines within the past 3 months (assessed independently by two psychiatrists, with arbitration by a third if needed). Additional exclusion criteria applied to both groups included pregnancy, malignancy, active infection, recent use of psychotropic medication, high risk of obstructive sleep apnea (STOP-Bang ≥3), and circadian rhythm disturbances (e.g., shift work).

Inclusion criteria for healthy controls were: (1) age 18–68 years (matched to cases); (2) no history of chronic diseases (e.g., hypertension, diabetes, cardiovascular disease); (3) no complaint of sleep disturbance (PSQI ≤5); (4) no anxiety (HAMA <14) or depression (PHQ-9 <10) confirmed by psychiatric evaluation (Supplementary material 1). Most questionnaires were completed during outpatient visits, with a minority collected via telephone or online survey (Supplementary Figure S1).

A total of 87 eligible ITP patients and 71 healthy controls were finally included. Written informed consent was obtained from all participants before enrollment. The study was conducted by the Declaration of Helsinki and relevant ethical guidelines and was approved by the Ethics Committee of Nanfang Hospital, Southern Medical University.

### Sample size estimation

The primary endpoint of this study was the difference in PSQI total score between ITP patients and healthy controls. A previous systematic review and meta-analysis of autoimmune diseases reported an average PSQI difference of approximately 3.0 points between patients and healthy populations (31). Considering the similarity of disease burden between ITP and other chronic inflammatory conditions, the expected between-group difference was conservatively set at 3.0, with a standard deviation of 3.5, based on prior PSQI validation studies (32). Under a two-sided *α* = 0.05 and 80% power, the minimum required sample size was 22 subjects per group. Allowing for a 15% dropout rate, the target sample size was approximately 26 subjects per group, totaling at least 52 participants.

In addition, to evaluate within-patient improvement of PSQI following treatment, this study referred to the reported minimal clinically important difference (MCID) of about 4.4 points (29), indicating that such an improvement would be clinically meaningful. Ultimately, 87 patients and 71 controls were enrolled, far exceeding the minimum requirement for the primary endpoint. However, for correlation analyses, the statistical power may still be limited for detecting small effect sizes.

### ITP treatment protocol

Patients with immune thrombocytopenia (ITP) were treated with glucocorticoids, and some additionally received a thrombopoietin receptor agonist (TPO-RA) (33).

### 5-HT collection

Participants underwent morning (07:00–08:00) fasting (≥8 h) venous blood collection (5 mL from the antecubital vein). One aliquot was placed in lithium heparin vacuum tubes (17 IU mL^−1^, Greiner Bio-One, Austria), centrifuged at 4 °C (3,000 × g, 10 min) to obtain plasma for 5-HT quantification. All samples were stored at −80 °C with minimized freeze–thaw cycles.

Plasma 5-HT levels were quantified using the ST/5-HT ELISA kit from Yuannuo Biotech (Cat. YJL10030-96T, Chengdu, China), with a detection range of 50–800 ng mL^−1^ and a sensitivity of 9.38 ng mL^−1^. All assays were performed in strict accordance with the manufacturer’s instructions, with both samples and standards analyzed in duplicate, and absorbance was measured at 450 nm using a microplate reader.

### Data collection and evaluation metrics

During the study period (June 2020 to December 2021), healthcare professionals assessed enrolled patients and guided them in completing a questionnaire on sleep-related factors (Supplementary material 2). The questionnaire included (1) general demographic questions (gender, age); (2) disease-related indicators [platelet count, diagnostic staging, and bleeding severity assessed using the ITP Bleeding Assessment Tool (ITP-BAT)]; and (3) sleep quality evaluation via the PSQI, which comprises seven components: subjective sleep quality, sleep latency, sleep duration, habitual sleep efficiency, sleep disturbances, use of sleep medications, and daytime dysfunction. Each component is scored 0 (good) to 3 (poor), with a global score range of 0–21 (a score >5 indicates sleep disorders). Primary outcomes were sleep quality (assessed by PSQI) and platelet count (measured by an automated hematology analyzer). Secondary outcomes included daytime dysfunction and quality of life (evaluated via specialized questionnaires; Supplementary material 3). Given the constraints of COVID-19 prevention, assessments were conducted using a hybrid model (in-person and remote), with data quality verified by high inter-rater consistency (ICC = 0.93). All participants underwent standardized psychiatric screening, including DSM-5 diagnostic criteria and HAMA/PHQ-9 scales, with eligibility restricted to those with HAMA ≤14 and PHQ-9 ≤10.

### Statistical analysis

Statistical analyses were performed using SPSS 26.0 (IBM Corp., Armonk, NY, United States) and R version 4.3.2 (R Foundation for Statistical Computing, Vienna, Austria). Normality of continuous variables was assessed with the Shapiro–Wilk test (*α* = 0.05). Normally distributed data are presented as mean ± standard deviation and compared using independent-sample t tests or one-way ANOVA, while non-normally distributed data are reported as median (IQR) and analyzed with the Mann–Whitney *U* test.

The linear association between platelet counts and PSQI total score was explored by Pearson correlation, with distributional trends visualized in two- and three-dimensional plots. To account for potential confounding, ordinary least squares (OLS) regression was applied to evaluate the independent effects of age and platelet count on PSQI and its subscales (e.g., sleep efficiency, daytime dysfunction, subjective sleep quality), reporting standardized coefficients (*β*), *p*-values, and *R*^2^. Analyses were conducted primarily with the stats and car packages in R.

For repeated measures across the 12-week follow-up, linear mixed-effects models (LMM) were constructed with Patient ID as a random effect and time, platelet, and their interaction (platelet × time) as fixed effects, using the lme4 package. Missing data were addressed through multiple imputation with predictive mean matching (*m* = 10), and pooled estimates were derived according to Rubin’s rules, implemented via the mice package.

Effect sizes were quantified using Cohen’s d (small: 0.20–0.49; medium: 0.50–0.79; large: ≥0.80), calculated with the effsize package. *Post hoc* power analysis, based on the observed effect size for PSQI total score (*d* = 0.89), indicated >95% statistical power to detect between-group differences. All tests were two-tailed, with statistical significance set at *p* < 0.05.

## Results

### Characteristics and baseline data of ITP patients

A total of 87 ITP patients and 71 healthy controls were enrolled (Table 1). The ITP group showed a female predominance (71.26%), with males accounting for 28.74%, while the control group had a similar gender distribution (females 74.65%, males 25.35%). Age distribution revealed a median age of 37 years (range: 18–68) in the ITP group, with the majority (60.92%) aged 18–44 years, whereas the control group had a median age of 40 years, demonstrating good age-matching with the case group. Clinically, 13.80% of ITP patients were newly diagnosed (<3 months), 33.33% were in the persistent phase (3–12 months), and 52.87% were in the chronic phase (>12 months). Platelet counts were predominantly low, with 68.97% of patients having counts ≤30 × 10^9^/L and only 11.49% exceeding 50 × 10^9^/L, indicating a high bleeding risk in most cases. Based on ITP-BAT bleeding grading, 59.77% showed no clinical bleeding, 39.08% had mild bleeding, and only 1.15% exhibited moderate bleeding, with no cases of severe bleeding observed. Overall, the case and control groups were well-matched in demographic variables, ensuring comparability for subsequent analyses.

**Table 1:** Characteristics and baseline data of ITP patients.

Characteristic	Result	*p*-value	SMD
Age [*N*, *M* (%)]	37 (18–68)	0.38	0.09
18–44 years	53 (60.92)		
45–59 years	26 (29.88)		
≥60 years	8 (9.20)		
Sex [*N* (%)]		0.65	0.07
Man	25 (28.74)		
Woman	62 (71.26)		
Phase of ITP [*N* (%)]			
<3 months	12 (13.80)		
3–12 months	29 (33.33)		
>12 months	46 (52.87)		
Platelet level			
≤30 × 10/L	60 (68.97)		
(31–50) × 10/L	17 (19.54)		
≥50 × 10/L	10 (11.49)		
ITP-BAT bleeding grading [*N* (%)]			
No bleeding	52 (59.77)		
Mild bleeding	34 (39.08)		
Morderate bleeding	1 (1.15)		
Massive bleeding	0 (0.00)		
Severe bleeding	0 (0.00)		

### Comparison of sleep quality between ITP patients and healthy individuals

**Figure 1:**
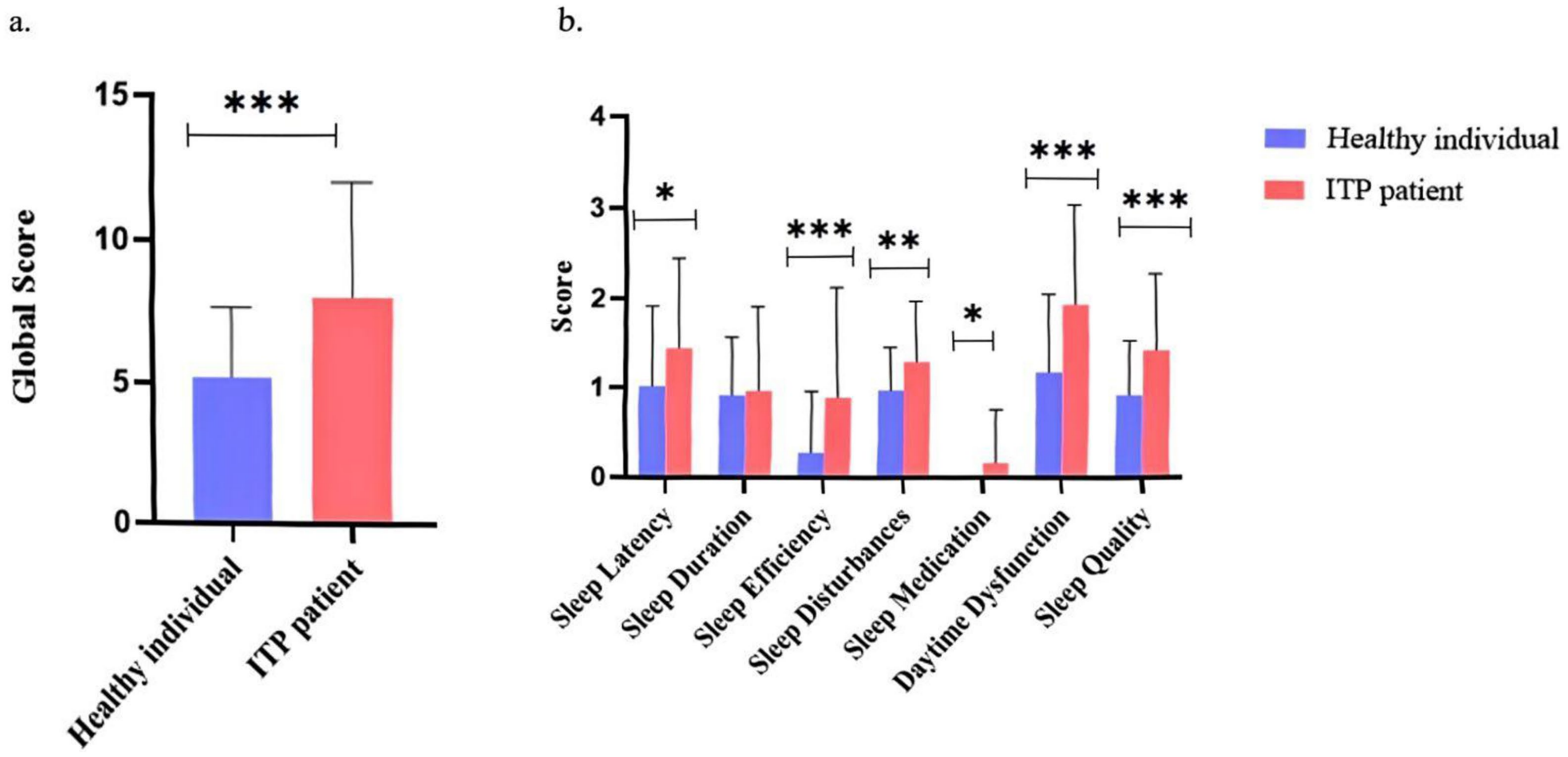
Comparison of sleep quality metrics between ITP patients and healthy controls. **(a)** Global score comparison showing overall sleep quality ratings between ITP patients and healthy controls. **(b)** Component-wise comparisons including sleep latency, sleep duration, sleep efficiency, sleep disturbance, use of sleep medication, daytime dysfunction, and subjective sleep quality. The study included 87 ITP patients (71.26% female, 28.74% male) aged 18 to ≥60 years. Analyses employed *t*-tests and ANOVA. Asterisks denote statistical significance (^*^p < 0.05, ^**^p < 0.01, and ^***^p < 0.001). Error bars represent standard deviation.

Compared with healthy controls, patients with ITP exhibited significantly poorer sleep quality (*p* < 0.001, Figure 1a). The PSQI total score in the ITP group was non-normally distributed (Shapiro–Wilk test, *p* = 0.02), with a median (IQR) of 8.0 (5.0–11.0), which was significantly higher than that of the control group [4.0 (2.0–6.0); Mann–Whitney *U* test, *p* < 0.001; effect size *r* = 0.42], indicating more severe sleep disturbances among ITP patients. Analysis of the seven PSQI components revealed significantly elevated scores in sleep latency (*p <* 0.05), sleep efficiency (*p <* 0.001), sleep disturbance (*p <* 0.01), daytime dysfunction (*p <* 0.001), and subjective sleep quality (*p <* 0.001) among ITP patients (Figure 1b). Effect size analysis showed medium-to-large effects for sleep efficiency (Cohen’s *d* = 0.65), medium effects for daytime dysfunction (*d* = 0.58) and subjective sleep quality (*d* = 0.52), and smaller but clinically meaningful effects for sleep latency (*d* = 0.41) and sleep disturbance (*d* = 0.39). While sleep duration and use of sleep medications showed slightly higher scores in ITP patients, these differences did not reach statistical significance. These findings collectively demonstrate that ITP patients experience significantly impaired sleep quality across multiple PSQI dimensions, with particularly notable deficits in sleep efficiency and daytime dysfunction that likely contribute substantially to their reduced HRQOL.

### Comprehensive assessment of sleep quality

Evaluation using the PSQI revealed a positive correlation between age and PSQI scores in ITP patients (*r* = 0.295, *p* = 0.006), indicating that older patients tended to report poorer sleep quality. FACIT-F scores were negatively correlated with PSQI scores (*r* = −0.42, *p* < 0.001), suggesting that poorer sleep quality was associated with greater fatigue. Similarly, SF-36 physical and mental function scores were negatively correlated with PSQI (physical: *r* = −0.38, *p* = 0.001; mental: *r* = −0.35, *p* = 0.003), reflecting associations between impaired sleep and diminished overall health functioning.

Platelet counts were inversely correlated with PSQI scores (*r* = −0.223, *p* = 0.04), consistent with poorer sleep quality in patients with lower platelet levels. No significant correlations were observed between PSQI and gender (*r* = −0.073, *p* = 0.50), disease duration (*r* = −0.009, *p* = 0.94), or bleeding scores (*r* = −0.061, *p* = 0.58) (Table 2). In the healthy control group (*n* = 71), age showed a weaker correlation with PSQI scores (*r* = 0.18, *p* = 0.12) (Supplementary Table S1), a trend consistent with previous large-scale epidemiological studies. These results suggest that age-related decline in sleep quality is detectable in the general population, and may be more evident in ITP patients.

**Table 2:** Correlations between sleep quality, age, platelet count, and clinical factors in ITP patients.

	Sex	Age	Platelet count	Bleeding grading	Disease course	Sleep global score
Sex	1					
Age	−0.073	1				
Platelet count	0.175	−0.215^*^	1			
Bleeding grading	−0.253^*^	0.214	−0.528^**^	1		
Disease course	0.014	0.110	−0.182	0.122	1	
Sleep global score	−0.073	0.295^**^	−0.223^*^	−0.061	−0.009	1

^*^p < 0.01; ^**^p < 0.05.

### Correlation between sleep quality and clinical indicators

**Figure 2:**
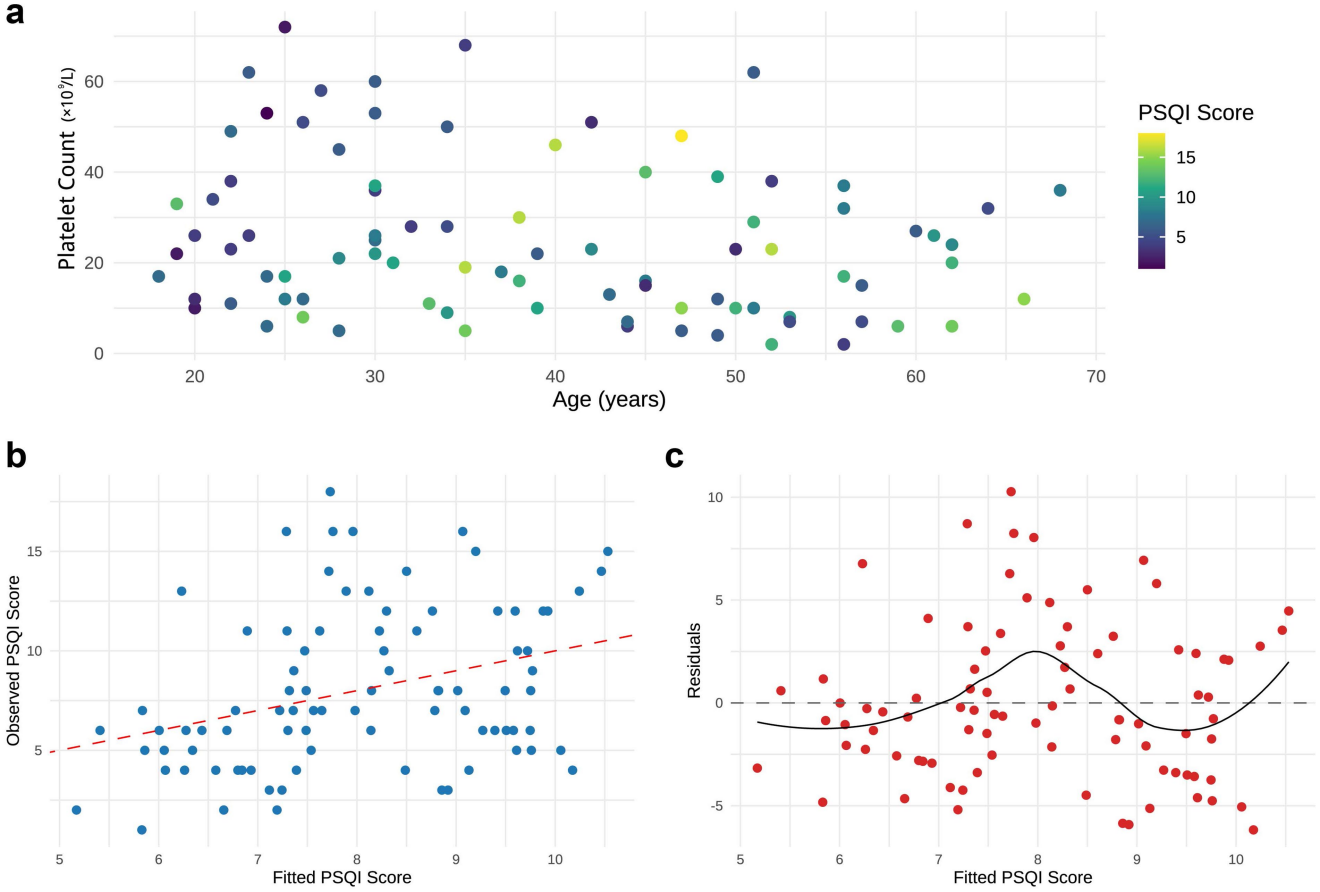
Linear analysis of age, platelet count, and sleep quality scores in ITP Patients. **(a)** Two-dimensional scatter plot of age versus platelet count, with point colors representing PSQI total scores (darker colors indicate poorer sleep quality). **(b)** Regression plot comparing observed PSQI scores with model-fitted values. **(c)** Residual plot of the model. All plots were generated using data from *n* = 87 ITP patients.

Bivariate analysis was performed to examine the associations among age, platelet count, and sleep quality. Because healthy controls were not assessed for platelet counts or disease severity, three-dimensional correlation plots were generated only for the ITP cohort. In ITP patients, PSQI total scores were positively correlated with age (*r* = 0.295, *p* = 0.006) and negatively correlated with platelet count (*r* = −0.223, *p* = 0.040), whereas no significant correlations were observed with gender, disease duration, or bleeding scores (Figure 2a).

In the multivariate linear regression model (PSQI ~ platelet + age), platelet count did not remain a significant predictor after adjusting for age (*β* = −0.039, *p* = 0.116). By contrast, age was retained as a significant predictor of PSQI scores (*β* = 0.074, 95% CI: 0.014–0.134, *p* = 0.016), and the model showed moderate explanatory power (*R*^2^ = 0.114, *p* = 0.006) (Figures 2b,c). These findings suggest that in the present dataset, age is the more consistent correlate of sleep quality among ITP patients, whereas platelet count shows only a modest, non-significant trend after adjustment.

### Characteristics of sleep disorders in specific age groups

**Figure 3:**
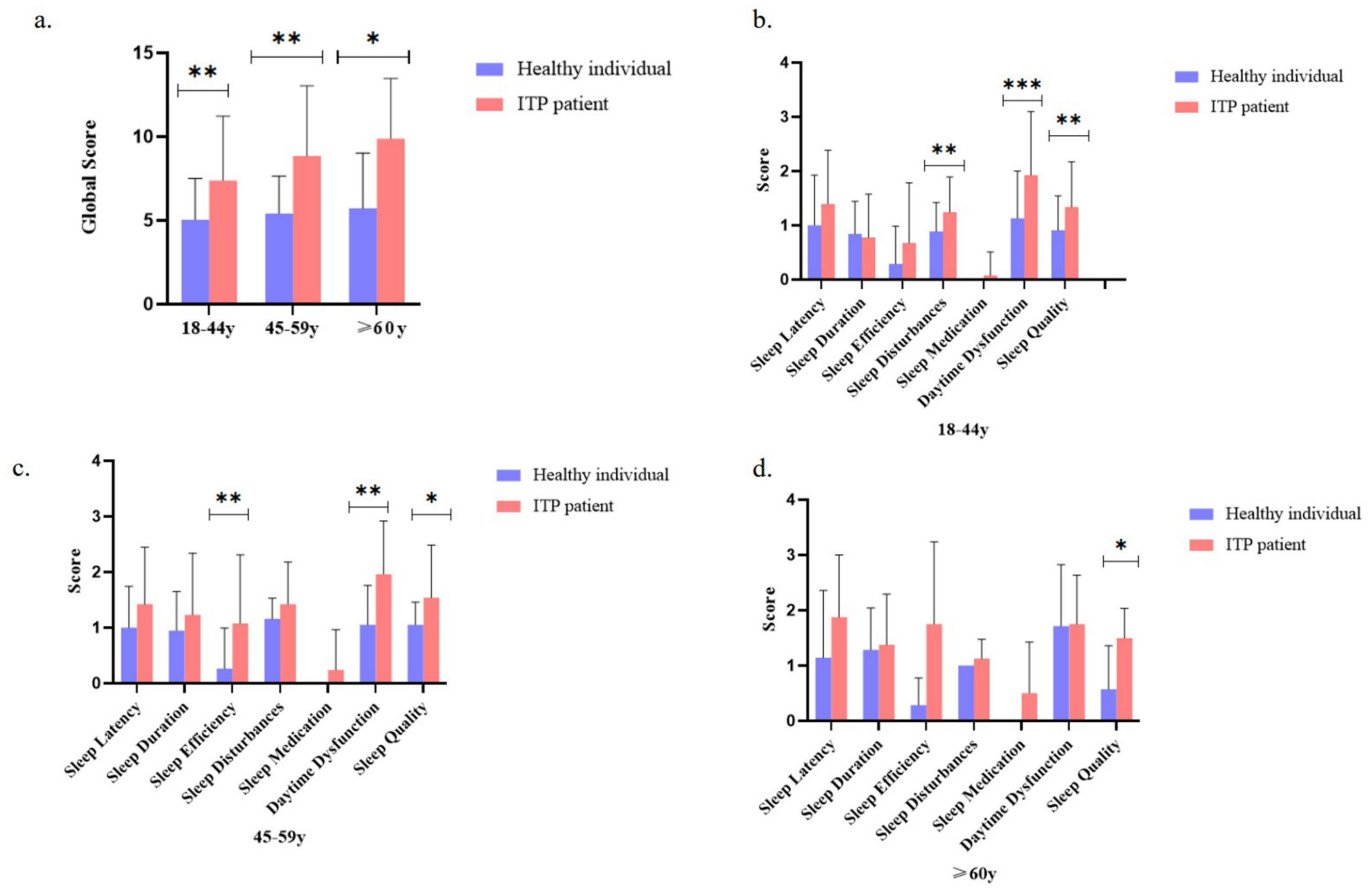
Age-stratified comparison of sleep quality metrics between ITP patients and healthy controls. **(a)** Comparison of global sleep quality scores between ITP patients and controls across age groups (18–44, 45–59, and ≥60 years). **(b)** Component-wise sleep parameter comparisons (sleep latency, duration, efficiency, disturbance, medication use, daytime dysfunction, and subjective quality) for 18–44-year-old ITP patients versus age-matched controls. **(c)** Same sleep parameter comparisons for the 45–59-year-old group. **(d)** Comparative analysis for the ≥60-year-old group. The study included 87 ITP patients analyzed by age stratification. Statistical significance was determined using *t*-tests and ANOVA, with asterisks denoting significance levels: ^*^p < 0.05, ^**^p < 0.01, and ^***^p < 0.001. Error bars represent standard deviation.

Subgroup analysis stratified by age revealed distinct patterns of sleep quality impairment in ITP patients (Figure 3). Compared with healthy controls, young (18–44 years) and middle-aged (45–59 years) ITP patients exhibited significantly higher PSQI global scores, whereas the difference diminished in patients ≥60 years (Figure 3a). In the 18–44-year group, scores for sleep disturbances, daytime dysfunction, and subjective sleep quality were significantly elevated, with the largest differences observed in daytime dysfunction and sleep disturbances (Figure 3b). Among patients aged 45–59 years, sleep efficiency and daytime dysfunction were significantly impaired relative to controls (Figure 3c). In contrast, for patients ≥60 years, only subjective sleep quality remained significantly different (Figure 3d). These results indicate that the impact of ITP on sleep quality varies by age group, with more pronounced differences observed in younger and middle-aged patients, while in older patients, only the subjective perception of sleep quality remained significantly altered.

### Impact of platelet count on sleep quality

**Figure 4:**
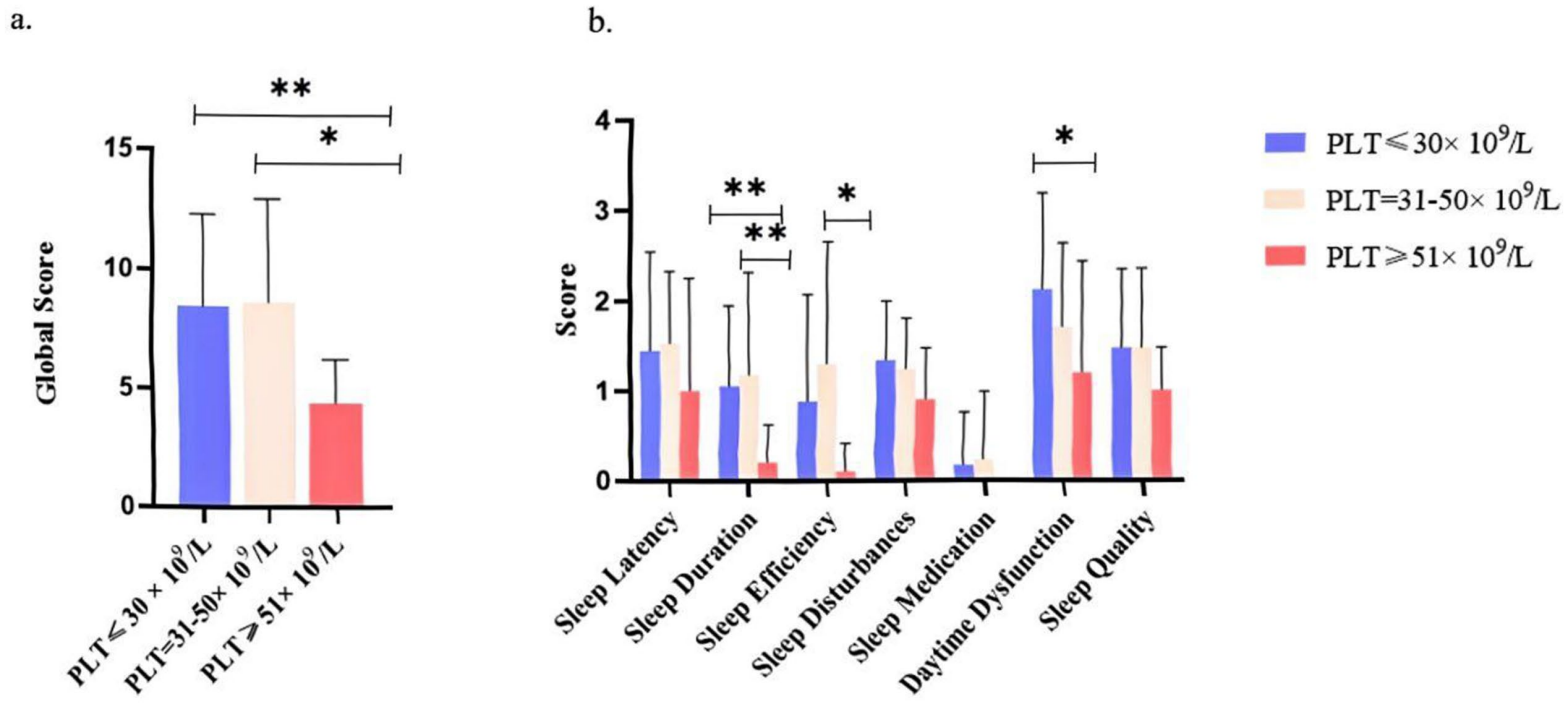
Sleep quality scores across platelet count subgroups in ITP Patients. **(a)** Global sleep quality scores of ITP patients stratified by platelet count (PLT ≤30 × 10^9^/L, PLT = 31–50 × 10^9^/L, PLT >50 × 10^9^/L). **(b)** Component-wise scores for specific sleep parameters (sleep latency, duration, efficiency, disturbance, medication use, daytime dysfunction, and subjective quality) across subgroups. The analysis included 87 ITP patients. Statistical significance was determined by *t*-tests and ANOVA (^*^p < 0.05 and ^**^p < 0.01). Error bars indicate standard deviation.

ITP patients were stratified into three subgroups based on platelet counts (≤30 × 10^9^/L, 31–50 × 10^9^/L, and >50 × 10^9^/L). Kruskal–Wallis analysis revealed significant group differences in global PSQI scores (*p* < 0.001), with *post hoc* comparisons showing that patients with platelet counts >50 × 10^9^/L had significantly lower PSQI scores compared to both the 31–50 × 10^9^/L and ≤30 × 10^9^/L groups (*q* < 0.01; Figure 4a). Subscale analysis further indicated significant group differences in sleep duration, sleep efficiency, and daytime dysfunction (all *p* ≤ 0.01), with a monotonic improvement observed across increasing platelet levels (*p*_trend ≤ 0.01; Figure 4b). Among these, daytime dysfunction differed most markedly between the >50 × 10^9^/L and ≤30 × 10^9^/L groups (*p* = 0.001, *q* < 0.01).

### Effects of platelet count and age on specific sleep parameters in ITP patients

**Figure 5:**
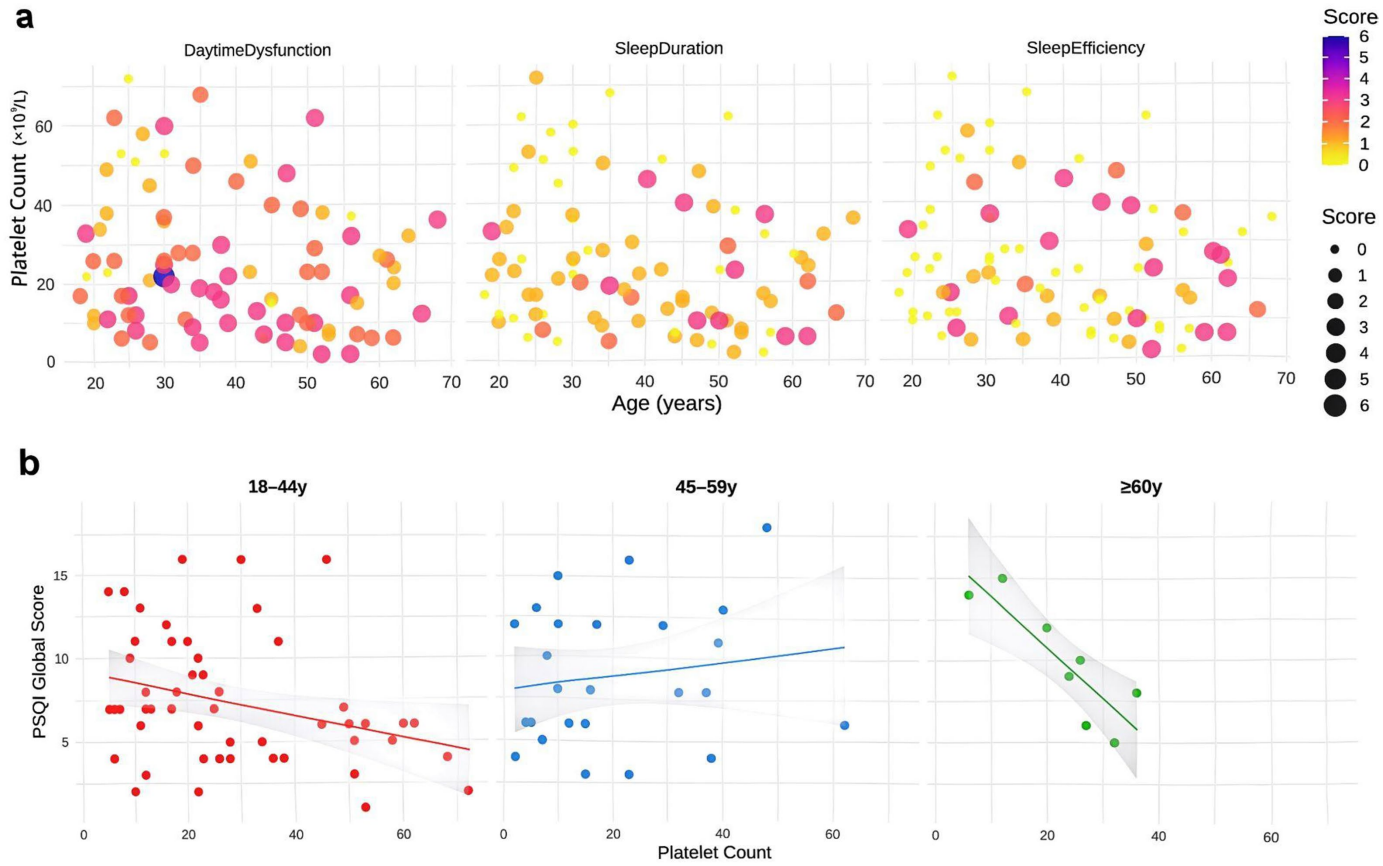
Analysis of platelet count and age effects on sleep parameters in ITP patients. **(a)** Scatter plots demonstrating joint effects of platelet count and age on sleep duration, efficiency, and daytime dysfunction scores. Point size reflects score intensity, with darker colors indicating worse scores. **(b)** Linear regression of platelet count versus PSQI total scores across age subgroups, with shaded 95% confidence intervals. Analyses were performed using data from 87 ITP patients.

Building upon these findings, we specifically analyzed the effects of platelet count and age on key sleep dimensions. Patients with lower platelet counts (<50 × 10^9^/L) consistently reported shorter sleep duration across all age groups (18–44, 45–59, ≥60 years; Figure 5a). Analysis of platelet-age interactions further revealed that the association between low platelet counts and poorer sleep efficiency became more pronounced with advancing age, particularly in the elderly (Figure 5b). Similarly, patients with platelet counts < 50 × 10^9^/L demonstrated higher levels of daytime dysfunction across nearly all age strata, indicating more severe daytime fatigue and concentration difficulties.

Beyond these primary parameters, analogous trends were observed across other PSQI components: patients with platelet counts <50 × 10^9^/L tended to report longer sleep latency, more sleep disturbances, increased use of sleep medications, and worse subjective sleep quality, although these differences did not reach statistical significance (Supplementary Figures S2a–d). Notably, the overall influence of age was relatively minor compared to platelet count, suggesting platelet levels may serve as a stronger biomarker for sleep quality disturbances in ITP patients.

Regression analyses of platelet count and PSQI total scores stratified by age further supported these findings. Significant negative associations were detected in both the 18–44 and ≥60 year groups, with the strongest effect in the elderly, whereas no significant linear relationship was observed in the middle-aged group (45–59 years; Figure 5b). Taken together, these results highlight that low platelet counts are associated with broad impairments in sleep quality, with the impact on sleep efficiency being exacerbated by aging.

### Improvement in sleep quality in ITP patients before and after treatment

**Figure 6:**
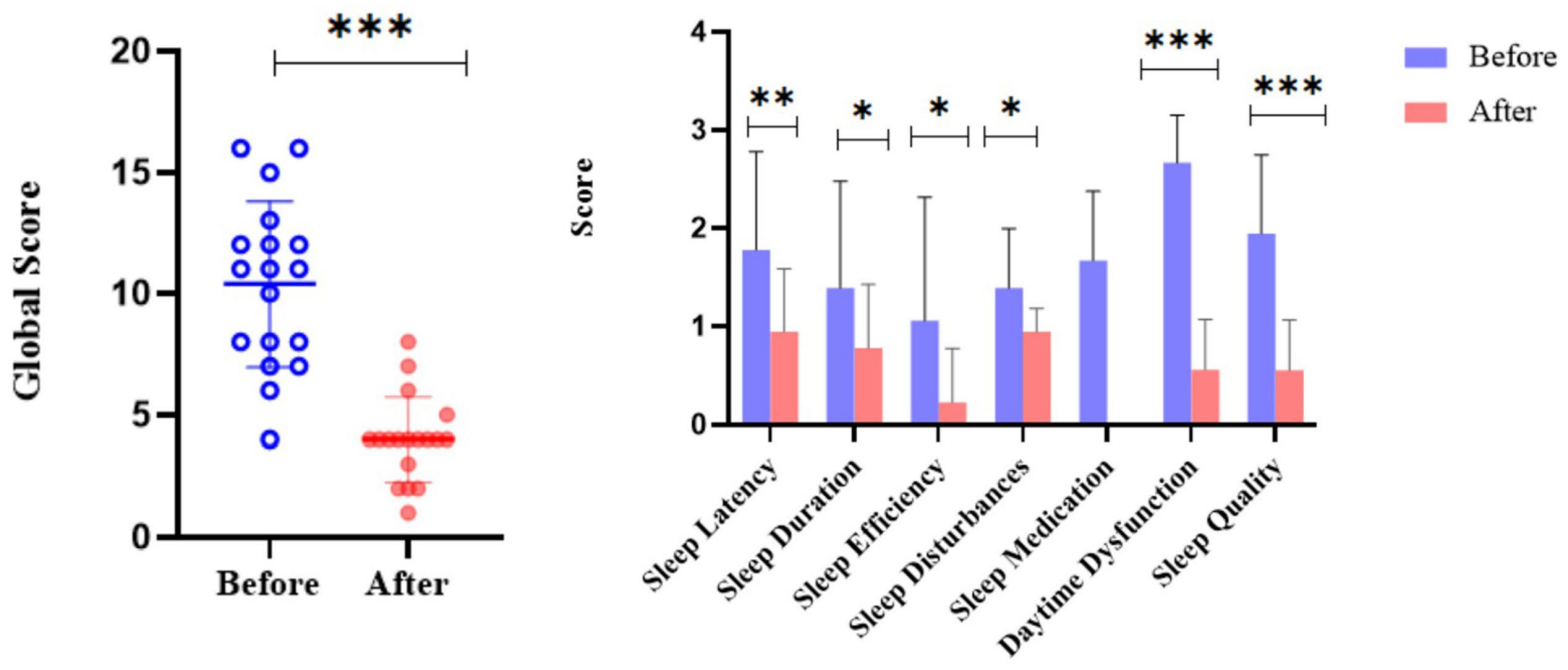
Comparison of sleep quality in ITP patients before and after treatment. The dot plot displays the distribution of global sleep quality scores pre- and post-treatment for individual patients. The adjacent bar chart illustrates score changes across specific sleep parameters (sleep latency, duration, efficiency, disturbances, medication use, daytime dysfunction, and subjective quality). The analysis included ITP patients with high baseline PSQI scores. Asterisks denote statistical significance levels: ^*^p < 0.05, ^**^p < 0.01, ^***^p < 0.001. Error bars represent standard error.

Pretreatment assessments revealed elevated scores across all sleep parameters, while post-treatment evaluations demonstrated significant reductions (*p <* 0.001, Figure 6), with notable improvements including shortened sleep latency (*p* = 0.004), increased sleep duration (*p* = 0.03), enhanced sleep efficiency (*p* = 0.01), and reduced sleep disturbances (*p* = 0.01, Figure 6). Post-treatment analysis also showed significant decreases in daytime dysfunction (*p <* 0.001) and subjective sleep quality scores (*p <* 0.001) (Figure 6). Patients achieving post-treatment platelet counts >50 × 10^9^/L exhibited significant increases in FACIT-F scores (Δ = 8.2 ± 3.1, *p <* 0.01) and SF-36 physical function scores (Δ = 7.5 ± 2.8, *p <* 0.01), confirming that therapeutic intervention not only ameliorated sleep parameters but also enhanced overall quality of life. These findings collectively demonstrate that targeted ITP treatment significantly improves multiple dimensions of sleep quality and general well-being.

## Discussion

This study demonstrated that patients with ITP had significantly higher PSQI scores than healthy controls, indicating impaired sleep quality, consistent with previous findings (19). Prior research has reported that female ITP patients generally experience worse sleep quality and fatigue compared with males, potentially due to estrogen-regulated inflammatory responses and psychological burden (10). In our analysis, the main sleep deficits in ITP patients were prolonged sleep latency, reduced sleep efficiency, increased sleep disturbances, daytime dysfunction, and poorer subjective sleep quality (Figures 3a–d). Psychological and physiological burdens, including concerns about bleeding risk, lifestyle restrictions, comorbidities, and fatigue, may further compromise sleep quality (34–36), as reflected by frequent nocturnal awakenings and vivid dreams (Supplementary Figure S3) as well as impaired daytime function. Moreover, reduced sleep quality was observed across all age groups in ITP patients compared with healthy individuals, with the most pronounced differences in younger adults (18–44 and 45–59 years), suggesting that psychological interventions and individualized treatment may be important adjuncts, particularly in younger patients.

Mechanistically, our findings highlight the potential role of the platelet-5-HT-melatonin axis. Serum 5-HT levels were significantly lower in ITP patients than in controls (*p* < 0.001, Supplementary Figure S4), indicating a storage defect due to reduced platelet counts. This deficiency may impair melatonin synthesis and circadian rhythm regulation (37–39). Experimental studies have shown decreased hippocampal 5-HT levels in ITP mouse models (40), while supplementation with 5-HT precursors improved fragmented sleep, further supporting the pathological role of this pathway. In addition, chronic inflammation in ITP, particularly elevated IL-6, can act on the hypothalamus through the blood–brain barrier, suppressing GABAergic neuronal activity and lowering arousal thresholds (41, 42). Platelet reduction may also enhance sympathetic activity and inhibit sleep-promoting nuclei such as the VLPO, thereby impairing sleep maintenance (43, 44). Furthermore, chronic microbleeding can lead to iron deficiency, reducing tyrosine hydroxylase activity and thereby impairing 5-HT synthesis and sleep regulation (45). Collectively, these findings suggest that multiple biological mechanisms drive sleep disturbance in ITP.

Longitudinal follow-up further demonstrated that effective treatment significantly improved sleep latency, duration, efficiency, sleep disturbances, daytime dysfunction, and subjective sleep quality (Figure 6). Higher 5-HT levels and improved sleep accompanied increases in platelet counts, and this association persisted after adjusting for glucocorticoid dosage, indicating that the relationship is independent of steroid side effects (46). Platelets are not only the main reservoir of circulating 5-HT (47, 48) but also contribute to inflammation by releasing platelet-derived microparticles (PMPs) containing cytokines such as IL-6 and TNF-α. Reduced 5-HT leads to decreased melatonin synthesis, which has been directly linked to shortened sleep duration and reduced efficiency (49, 50). Previous studies confirm that 5-HT is a key sleep-promoting neurotransmitter (51, 52), and that total sleep time, sleep efficiency, and subjective sleep quality correlate positively with melatonin levels (53). Our results (Supplementary Figure S4) therefore support the hypothesis that reduced 5-HT and its downstream metabolite melatonin underlie impaired sleep in ITP patients.

Further analysis revealed a nonlinear relationship between platelet counts and sleep quality: PSQI deterioration was most pronounced when PLT <50 × 10^9^/L, while the effect diminished at higher levels, suggesting a threshold effect. In multivariate models, age emerged as a stronger predictor than platelet count. Stratified and interaction analyses showed that elderly patients (≥60 years) were more susceptible to the adverse sleep effects of thrombocytopenia. This may be explained by two mechanisms: first, platelet 5-HT storage capacity declines with age (54), and melatonin secretion is also reduced in the elderly (55), exacerbating sleep disruption when platelet counts fall; second, inflammaging places older adults in a chronic proinflammatory state, while ITP patients also exhibit elevated IL-6 (56), and inflammatory signals have been closely linked to sleep disturbances (57). These findings suggest that both the platelet-5-HT-melatonin axis and inflammaging mechanisms may act synergistically, explaining the greater vulnerability of elderly ITP patients.

This study reveals a potential biological pathway whereby platelet reduction leads to peripheral 5-HT and melatonin deficiency, while an inflammation-senescence state further aggravates the process, jointly contributing to sleep disturbances. The findings highlight the particular vulnerability of elderly ITP patients. Clinically, special attention should be paid to patients aged ≥60 years with PLT <50 × 10^9^/L, in whom screening and management of sleep disorders are warranted in addition to bleeding risk. The clinical implications are threefold: (1) sleep quality is negatively correlated with platelet count, suggesting that patients with severe thrombocytopenia face a higher risk of sleep impairment; (2) potential interventions may include therapies that increase circulating 5-HT/melatonin as well as psychological support and individualized management, especially for younger patients; and (3) the results provide a new perspective for comprehensive ITP care, emphasizing the integration of both physical and psychological health.

Methodologically, this prospective cohort combined longitudinal self-control with parallel healthy controls, providing dual advantages. The healthy control group not only confirmed the baseline abnormalities in ITP patients’ sleep quality but also quantified the natural variability of PSQI in the general population, serving as a benchmark for evaluating treatment-specific effects. The study adhered strictly to STROBE principles of temporal precedence (median interval between ITP diagnosis and assessment: 4.2 years), and ensured comparability through age/sex matching (SMD <0.1) and multivariable adjustment. Importantly, the introduction of healthy controls allowed for the establishment of an objective threshold for treatment response (PSQI reduction >1.0 point), thereby reinforcing the practical significance of the findings.

Several limitations should be acknowledged. First, the cross-sectional design precludes definitive causal inference regarding whether ITP leads to impaired sleep or vice versa. Second, the single-center cohort may limit generalizability. Third, the PSQI, while widely validated, relies on self-report and is subject to reporting bias, with no objective sleep measures included. Finally, the impact of different treatment strategies on sleep was not explored, restricting a comprehensive evaluation of therapeutic effects. In interpreting the findings, two factors must be weighed: the methodological rigor and the biological plausibility of the platelet-5-HT relationship support reliability, yet the study’s conduct during the pandemic raises the possibility of unmeasured confounders such as healthcare accessibility.

Future research should address these limitations through multi-center, large-sample longitudinal studies to clarify causal relationships between ITP and sleep disturbances. Incorporating objective sleep monitoring tools (e.g., actigraphy) would reduce reliance on self-report. Further work should systematically examine the differential effects of ITP treatments on sleep and evaluate novel interventions targeting 5-HT/melatonin metabolism or anti-inflammatory pathways. Ultimately, these efforts may lead to more comprehensive management strategies that not only improve platelet counts but also enhance sleep quality and overall quality of life in ITP patients. Replication in non-pandemic settings will be crucial to confirm the generalizability and stability of the findings.

In summary, this study provides an in-depth investigation of the impact of ITP on patients’ sleep quality and examines the therapeutic effects on sleep improvement. The results demonstrate significant differences between ITP patients and healthy controls across multiple sleep parameters, particularly in sleep latency, sleep efficiency, sleep disturbances, and daytime dysfunction. Correlation analyses identified age and platelet count as key factors influencing sleep quality, with lower platelet counts showing a negative correlation with sleep impairments. Following treatment, ITP patients with initially high PSQI scores exhibited significant overall sleep quality improvements, most notably in sleep latency, duration, efficiency, and disturbance measures. These findings suggest that sleep disturbances in ITP patients may be closely associated with disease status and that targeted treatment can substantially enhance both sleep quality and overall quality of life.

## Data Availability

The original contributions presented in the study are included in the article/Supplementary material, further inquiries can be directed to the corresponding author.

## Supplementary material

SUPPLEMENTARY FIGURE S1 Flowchart of patient screening and randomization process for ITP Study.

SUPPLEMENTARY FIGURE S2 Specific effects of platelet count and age on sleep latency, sleep disturbances, sleep medication use, and subjective sleep quality. **(a,b,d)** Patients with platelet counts <50 × 10^9^/L showed higher scores for sleep latency, sleep disturbances, and subjective sleep quality across all age groups. **(c)** For sleep medication use, patients >30 years old with platelet counts <50 × 10^9^/L demonstrated higher scores (this finding may not be representative due to limited sample size and generally low usage of sleep medications among patients).

SUPPLEMENTARY FIGURE S3 Specific contributing factors to sleep disturbances in sleep deficiency.

SUPPLEMENTARY FIGURE S4 Circulating 5-HT levels in healthy controls versus itp patients. ITP patients exhibited significantly lower 5-HT levels compared to healthy controls (^**^*p <* 0.01).
