# Supplementary figures and images for: Platelet count and sleep quality in immune thrombocytopenia: correlation with 5-hydroxytryptamine and therapeutic implications of platelet-5-HT-melatonin axis dysregulation
Source: Front Neurol. 2025 Oct 20;16:1645796. doi: 10.3389/fneur.2025.1645796 (PMC12593468; doi:10.3389/fneur.2025.1645796)

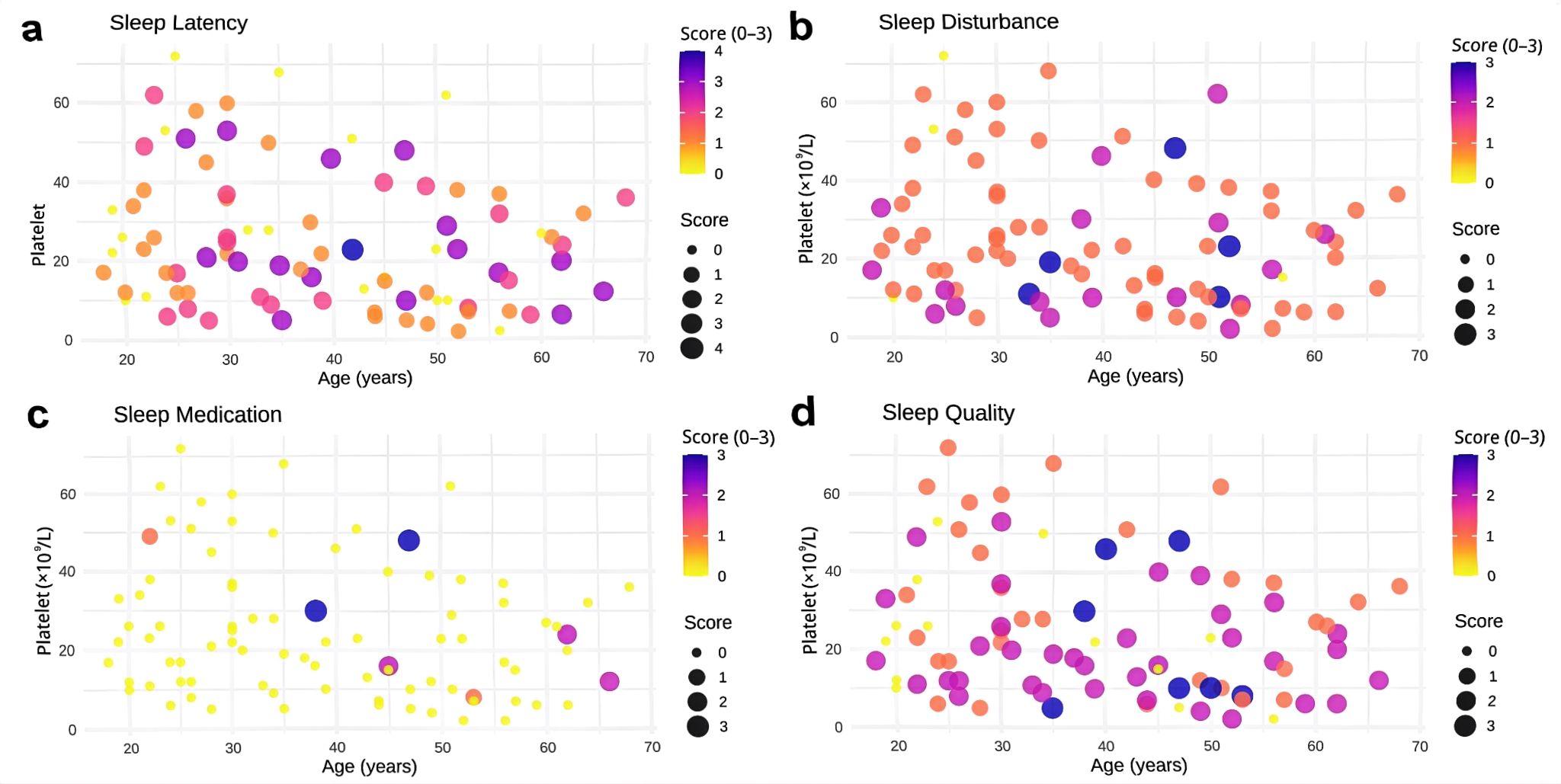

Supplement: SUPPLEMENTARY FIGURE S2 — Specific effects of platelet count and age on sleep latency, sleep disturbances, sleep medication use, and subjective sleep quality. (a,b,d) Patients with platelet counts <50 × 109/L showed higher scores for sleep latency, sleep disturbances, and subjective sleep quality across all age groups. (c) For sleep medication use, patients >30 years old with platelet counts <50 × 109/L demonstrated higher scores (this finding may not be representative due to limited sample size and generally low usage of sleep medications among patients). [file Image_2.jpeg]

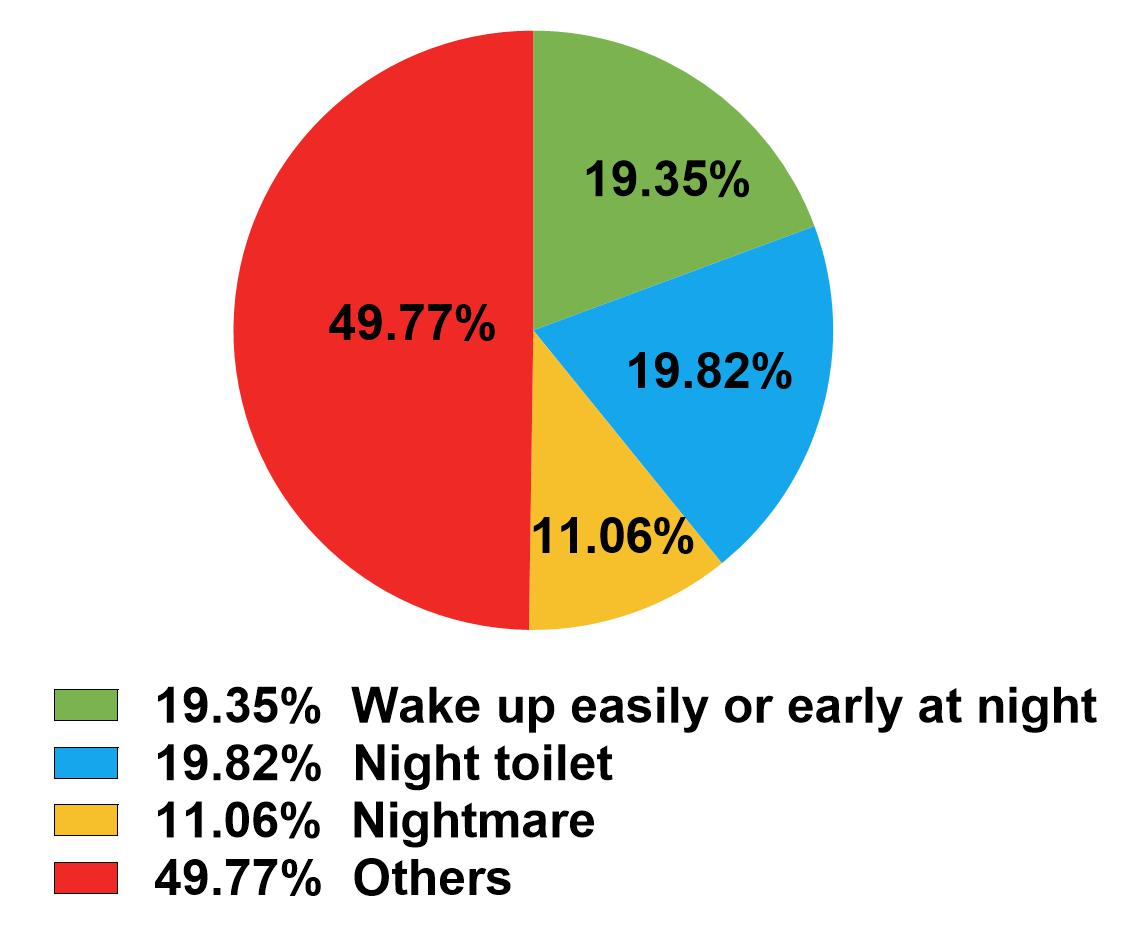

Supplement: SUPPLEMENTARY FIGURE S3 — Specific contributing factors to sleep disturbances in sleep deficiency. [file Image_3.jpeg]

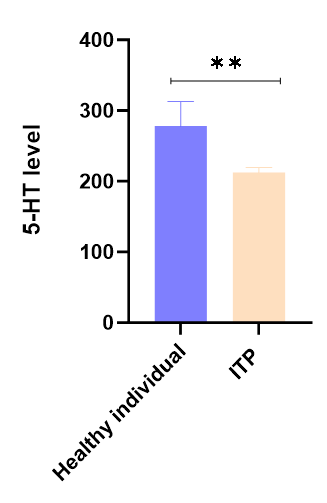

Supplement: SUPPLEMENTARY FIGURE S4 — Circulating 5-HT levels in healthy controls versus itp patients. ITP patients exhibited significantly lower 5-HT levels compared to healthy controls (**p < 0.01). [file Image_4.tif]
