# Supplementary material for: Platelet count and sleep quality in immune thrombocytopenia: correlation with 5-hydroxytryptamine and therapeutic implications of platelet-5-HT-melatonin axis dysregulation
Source: Front Neurol. 2025 Oct 20;16:1645796. doi: 10.3389/fneur.2025.1645796 (PMC12593468; doi:10.3389/fneur.2025.1645796)
Supplement: Supplementary file 6 [file Table_2.docx]

1. **Hamilton Anxiety Scale (HAMA)**
   **Description:** The scale consists of 14 items and is clinician-rated. Each item is scored on a scale from 0 to 4, yielding a total score ranging from 0 to 56. A total HAMA score >14 indicates moderate to severe anxiety and is one of the exclusion criteria in this study.

| **Item** | **Score (0–4)** |
| --- | --- |
| 1. Anxious mood (worry, anticipation of the worst) |  |
| 2. Tension (irritability, inability to relax) |  |
| 3. Fears (of the dark, of strangers, of being alone, of animals, etc.) |  |
| 4. Insomnia |  |
| 5. Cognitive impairment (poor concentration, memory problems) |  |
| 6. Depressed mood |  |
| 7. Muscular symptoms (aches, fatigue, tremor) |  |
| 8. Sensory symptoms (tinnitus, blurred vision, headaches) |  |
| 9. Cardiovascular symptoms (palpitations, chest tightness) |  |
| 10. Respiratory symptoms (shortness of breath, chest tightness) |  |
| 11. Gastrointestinal symptoms (nausea, bloating, diarrhea) |  |
| 12. Genitourinary symptoms (frequent urination, decreased libido) |  |
| 13. Autonomic symptoms (dry mouth, sweating) |  |
| 14. Behavior at interview (restlessness, fidgeting) |  |

1. **Patient Health Questionnaire-9 (PHQ-9)**
   **Description:** This self-administered questionnaire consists of 9 items. Each item is rated from 0 to 3, with a total score ranging from 0 to 27. A PHQ-9 total score >10 indicates moderate to severe depression and serves as one of the exclusion criteria for this study.

| **Item** | **Score (0–3)** |
| --- | --- |
| 1. Little interest or pleasure in doing things |  |
| 2. Feeling down, depressed, or hopeless |  |
| 3. Trouble falling or staying asleep, or sleeping too much |  |
| 4. Feeling tired or having little energy |  |
| 5. Poor appetite or overeating |  |
| 6. Feeling bad about yourself — or that you are a failure or have let yourself or your family down |  |
| 7. Trouble concentrating on things, such as reading the newspaper or watching television |  |
| 8. Moving or speaking so slowly that other people could have noticed, or being so fidgety or restless that you have been moving a lot more than usual |  |
| 9. Thoughts that you would be better off dead or of hurting yourself in some way |  |
