# Supplementary material for: Platelet count and sleep quality in immune thrombocytopenia: correlation with 5-hydroxytryptamine and therapeutic implications of platelet-5-HT-melatonin axis dysregulation
Source: Front Neurol. 2025 Oct 20;16:1645796. doi: 10.3389/fneur.2025.1645796 (PMC12593468; doi:10.3389/fneur.2025.1645796)
Supplement: Supplementary file 7 [file Table_3.docx]

# Sleep Quality and Bleeding Assessment Questionnaire

Dear Participant:
Thank you for participating in this study. This questionnaire is designed to collect information related to your sleep quality and bleeding status. Please fill in the information based on your actual condition. All data will be used strictly for research purposes, and your information will remain confidential.

## I. General Information

1. Name: ___________________________

2. Gender:
 [ ] Male
 [ ] Female

3. Age: ___________ years old

4. Disease duration: ___________ years

## II. Sleep Quality Assessment (Pittsburgh Sleep Quality Index, PSQI)

Please fill out the following items based on your sleep condition over the past month.

1. Sleep Latency
How long does it usually take you to fall asleep after lying down?
[ ] 0 (Less than 15 minutes)
[ ] 1 (15-30 minutes)
[ ] 2 (30-60 minutes)
[ ] 3 (More than 60 minutes)

2. Sleep Duration
How many hours of sleep do you get on average each night?
[ ] 0 (More than 7 hours)
[ ] 1 (6-7 hours)
[ ] 2 (5-6 hours)
[ ] 3 (Less than 5 hours)

3. Sleep Efficiency
What is the ratio of actual sleep time to the time spent in bed?
[ ] 0 (More than 85%)
[ ] 1 (75%-84%)
[ ] 2 (65%-74%)
[ ] 3 (Less than 65%)

4. Sleep Disturbances
Did you experience interruptions or problems with your sleep in the past month?
[ ] 0 (None)
[ ] 1 (Occasionally)
[ ] 2 (Sometimes)
[ ] 3 (Frequently)

5. Use of Sleep Medication
Did you use any medication to help you sleep in the past month?
[ ] 0 (None)
[ ] 1 (Less than once a week)
[ ] 2 (1-2 times per week)
[ ] 3 (3 or more times per week)

6. Daytime Dysfunction
How often did sleep problems interfere with your daytime work or daily activities?
[ ] 0 (None)
[ ] 1 (Occasionally)
[ ] 2 (Sometimes)
[ ] 3 (Frequently)

7. Subjective Sleep Quality
How would you rate the quality of your sleep in the past month?
[ ] 0 (Very good)
[ ] 1 (Fairly good)
[ ] 2 (Fairly bad)
[ ] 3 (Very bad)

## III. Bleeding Assessment

1. Platelet Count (PLT): __________ × 10^9^/L

2. Bleeding Condition (Please check all that apply):
[ ] None
[ ] Bruises
[ ] Petechiae
[ ] Ecchymosis
[ ] Gum bleeding
[ ] Other (please specify): __________________________

## IV. Comprehensive Evaluation

1. Total Sleep Score (to be filled by the researcher): ___________

2. Overall Sleep Quality Assessment:
[ ] Very Good
[ ] Fairly Good
[ ] Fairly Bad
[ ] Very Bad

Please complete the questionnaire carefully. Thank you for your cooperation! If you have any questions, please consult the medical staff.
