# Supplementary material for: Platelet count and sleep quality in immune thrombocytopenia: correlation with 5-hydroxytryptamine and therapeutic implications of platelet-5-HT-melatonin axis dysregulation
Source: Front Neurol. 2025 Oct 20;16:1645796. doi: 10.3389/fneur.2025.1645796 (PMC12593468; doi:10.3389/fneur.2025.1645796)
Supplement: Supplementary file 8 [file Table_4.docx]

**Daytime Functional Impairment and Quality of Life Assessment Questionnaire for Patients with ITP**

**Section I: Demographic and Clinical Information**

1. Gender: □ Male □ Female
2. Age: ______ years
3. ITP Duration: □ Newly diagnosed (<3 months) □ Persistent (3–12 months) □ Chronic (>12 months)
4. Current platelet count: ______ ×10⁹/L

**Section II: Daytime Functional Impairment Assessment**

(Adapted from the Epworth Sleepiness Scale and Fatigue Severity Scale)

Please rate each item according to your actual condition over the past month:
0 = Never, 1 = Rarely, 2 = Sometimes, 3 = Often, 4 = Always

1. Do you feel fatigued or lack energy during the day?
2. Do you experience difficulty concentrating or memory decline during the day?
3. Does fatigue frequently interrupt your work or study?
4. Do you require naps or prolonged rest to maintain daily activities?
5. Do you feel dizzy or drowsy during the day?
6. Are your social activities (e.g., gatherings, sports) limited due to fatigue?
7. Do you reduce daily activities due to concerns about bleeding risk?

**Section III: Quality of Life Assessment**

(Adapted from the SF-36 and ITP-specific QoL scales)

Please rate each item based on your actual condition over the past month:
0 = Not at all, 1 = A little, 2 = Moderate, 3 = Quite a lot, 4 = Very much

**Physical Functioning Domain**

1. Have you had difficulty completing daily chores due to fatigue or weakness?
2. Have you avoided strenuous physical activity due to dizziness or fear of bleeding?
3. Have you reduced your working hours or study time due to illness-related limitations?

**Psychological Functioning Domain**

1. Have you felt anxious or depressed due to recurrent disease episodes?
2. Are you worried about potential serious bleeding complications in the future?
3. Has your mood been affected by treatment side effects (e.g., corticosteroid-induced emotional changes)?

**Social Functioning Domain**

1. Have you reduced interactions with friends or family due to the disease?
2. Have you avoided long-distance travel or outings due to fatigue or bleeding risk?

**Section IV: Bleeding-Related Symptoms**

(Simplified from the ITP Bleeding Assessment Tool [ITP-BAT])

Please record the frequency of bleeding events in the past month:

- Skin bruising or petechiae: □ None □ Occasional (<1 time/month) □ Frequent (≥1 time/month)
- Nosebleeds: □ None □ Occasional □ Frequent
- Gum bleeding: □ None □ Occasional □ Frequent
- Heavy menstrual bleeding (for females): □ None □ Mild □ Severe

**Scoring and Interpretation**

1. Daytime Functional Impairment Score: 0–28 points; higher scores indicate greater functional impairment.
2. Quality of Life Score: 0–32 points; higher scores indicate more severely impaired quality of life.
3. Bleeding Symptom Score: 0–4 points; scores ≥2 suggest a need for enhanced hemostatic management.
